# Coproduced resources to support parents caring for children with gastrostomies

**DOI:** 10.1136/flgastro-2022-102181

**Published:** 2022-08-25

**Authors:** Bethan Page, Emily Harrop, Tania Beale, Katherine Boyce, Colette Smith, Siobhan Butler, Alison Sharrard, Charles Vincent, Alex CH Lee

**Affiliations:** 1 Department of Experimental Psychology, University of Oxford, Oxford, UK; 2 Helen and Douglas House, Oxford, UK; 3 Community Children's Nursing, Oxford Health NHS Foundation Trust, Oxford, UK; 4 Department of Paediatrics, Oxford University Hospitals NHS Foundation Trust, Oxford, UK

**Keywords:** gastrostomy, paediatric gastroenterology, health service research

## Abstract

**Objective:**

To describe and disseminate a package of support for parents who care for children with gastrostomies, consisting of a library of videos and resources to support families from referral for gastrostomy surgery, to long-term support at home.

**Methods:**

The resources were systematically developed and evaluated by parents, hospital and community-based nurses, paediatricians, a surgeon and researchers.

**Results:**

The videos empower families, reduce their anxiety and increase their confidence, providing support throughout the families’ journey. Surveys and feedback from parents and clinicians show that the video library is seen as providing clear and comprehensive guidance and is suitable for integration into routine practice. To effectively disseminate these resources across a region, the videos need to be shared widely with relevant community and hospital-based teams, and shared through parent networks. The videos should be viewed as one part of a wider package of training and support, in combination with hands-on-practice and clinical support.

**Conclusions:**

The resources described have been developed with and for families. Critically the videos are founded in the lived-experience of families, as well as the expertise of clinicians from community and hospital services. Similar resources are needed to support families performing other types of specialist care. The resources are freely available to any parent or clinical team.

**WHAT IS ALREADY KNOWN ON THIS TOPIC**

- Training and support for families caring for children with gastrostomies often does not fully meet families’ needs.
- Inadequate training and support for families can lead to avoidable harm to children and unnecessary visits to hospital or callouts to community teams.
- Families are often very anxious about caring for their child’s gastrostomy, especially in the immediate postoperative period.

**WHAT THIS STUDY ADDS**

- We have developed a library of videos and online resources to support parents caring for children with gastrostomies. The resources help families from referral for a gastrostomy to long-term support in the community.
- The resources have been coproduced with families and specialist healthcare professionals from across hospital and community services.
- A formal evaluation of the videos with clinicians and families shows that they are seen as providing clear and comprehensive guidance and are suitable for integration into routine practice. The aim of this paper is to help disseminate the resources and provide advice on implementation.

**HOW THIS STUDY MIGHT AFFECT RESEARCH, PRACTICE OR POLICY**

- The videos empower families and reduce their anxiety, providing support throughout the families’ journey.
- The resources may help reduce the demand on healthcare services, for example, unnecessary visits to Accident & Emergency (A&E) or callouts to community teams, and potentially the number of gastrostomy-related complications in children.
- The videos can help families to ask better questions in clinic and increase their confidence caring for their child.

## Introduction

Comprehensive training and support is needed to prepare parents to care for their child’s gastrostomy. Parents are required to learn how to administer feeds and medications, clean and care for the stoma site, perform maintenance tasks such as changing the water in a gastrostomy button and manage problems such as sore and leaking stoma sites, granulation tissue and blocked tubes. In this article, we report on a library of video resources coproduced by families and clinicians to support parents who care for children with gastrostomies, right from the initial referral for a gastrostomy to long-term support at home. The videos cover routine care of a gastrostomy button, troubleshooting common issues, other families’ experiences and information about the surgery itself. The resources were developed by a team of clinicians from across hospital and community services, alongside a team of researchers and parent representatives. Families were involved from the start of the project to ensure the resources fully addressed their needs. This article provides a brief summary of the development of the videos and their impact on families and services, before providing advice on how to embed these resources into routine practice. The primary aim of this article is to help disseminate these resources for the benefit of other clinical teams, patients and families.

### The need for better training and support for families

In an analysis of incident reports from England and Wales, healthcare professionals frequently reported concerns relating to inadequate training for families caring for children with enteral feeding devices, with cases of serious harm to children reported.^1^ In a survey of 146 UK families who care for a child with a gastrostomy, families reported that the training they received was often very brief and did not fully meet their needs.^2^ Many families reported feeling anxious about caring for their child’s gastrostomy, and stated that they would value more preparation and support.^2^ Parents report that teaching during the hospital admission is often rushed and too brief, with pressure to discharge families quickly.^2^ Community teams often lack the time, resources or expertise to provide the comprehensive training and support families need before and after the gastrostomy insertion. Many hospitals have reduced the length of stay for the procedure to 24–48 hours (or less), which means there is very limited time during the hospital admission to provide training. Reducing the length of stay for gastrostomy surgery has many advantages for hospitals and families; however, it does necessitate more training and information for families preoperatively.^3^ Comprehensive training for families is critical for optimising outcomes for children.^4^ There is a need to develop a multidisciplinary and cross-organisation approach to training and supporting families, which empowers families and ensures their children receive high-quality care at home.

## Development of the video library

**Figure 1:**
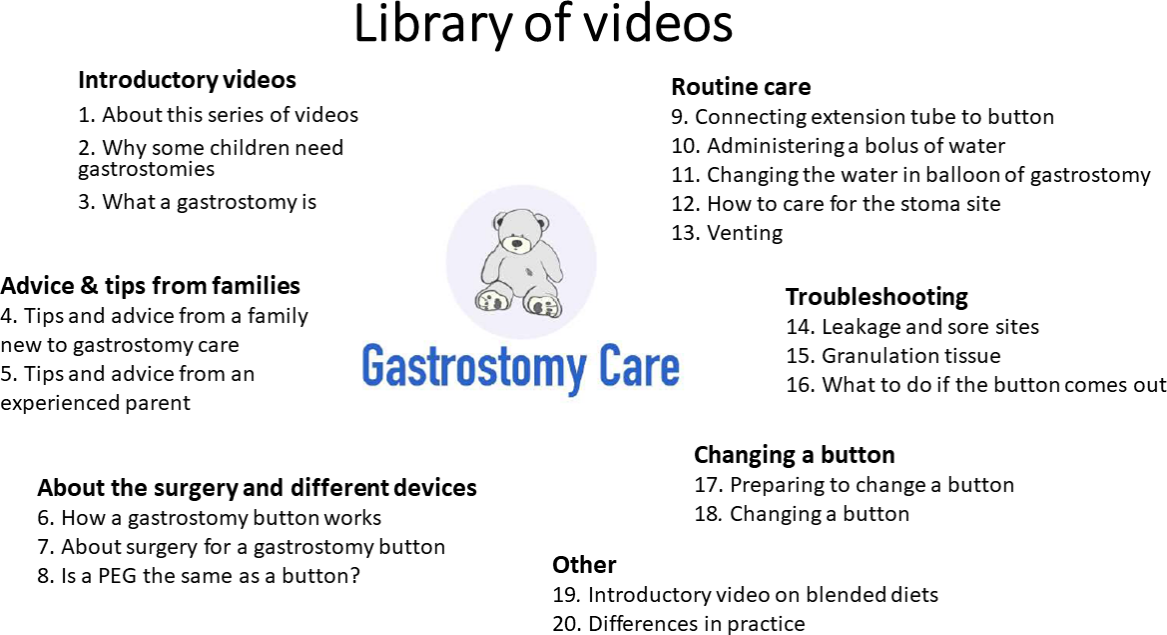
Library of videos illustrating the different topics included.

**Figure 2:**
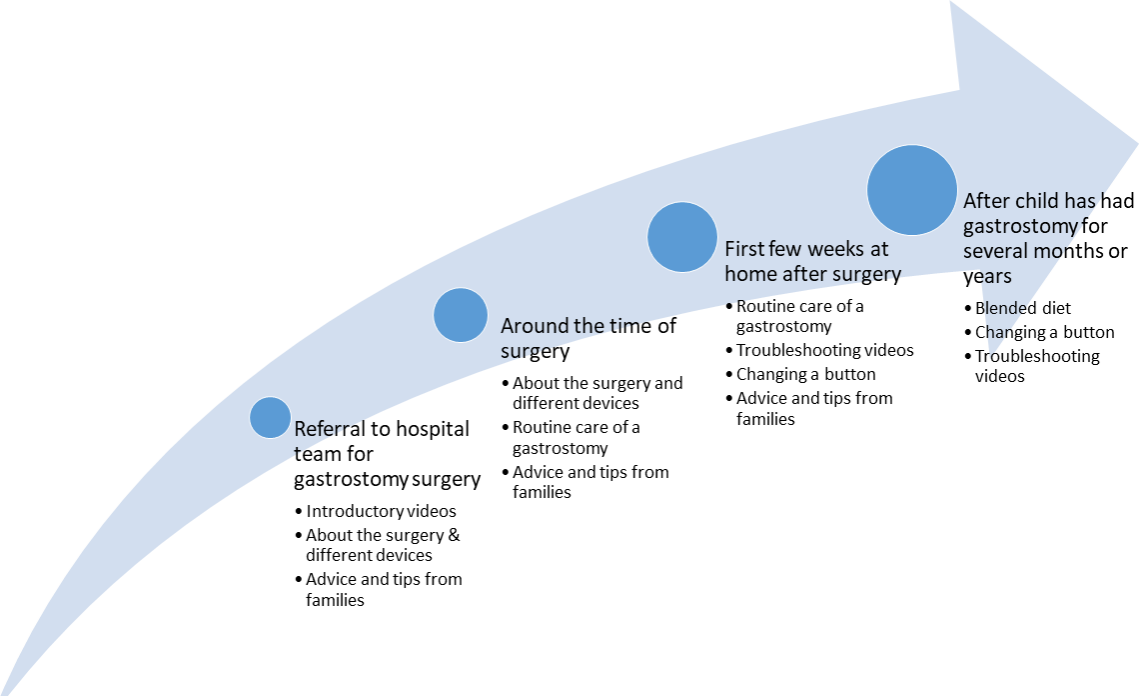
Diagram illustrating how the video topics fit within the patient pathway. It shows when the different videos will be most helpful for families to watch based on feedback from clinicans and families.

The video library was developed by a stakeholder group of parents, paediatricians, hospital and community-based nurses, a surgeon and researchers from the UK. There are 20 videos in the library. A full list of videos included is given in figure 1. The videos support families from when their child is referred for a gastrostomy, through to the surgery itself, to the immediate postoperative period and long-term support at home. Having a gastrostomy fitted is often a major life-changing decision for families.^5^ The resources are designed to address parents’ practical information needs but also to offer reassurance and emotional support to families. The videos feature a number of different parents and children, a specialist nurse, community nurses, a surgeon and a paediatrician. In some of the videos, the information is copresented by families and clinicians. Some videos feature families caring for their children in the home environment, which was emphasised as critical by our parent representatives. A full evaluation of the videos and more information on the development process is available elsewhere.^6^ Figure 2 illustrates how the videos fit within the existing patient pathway based on feedback from the formal evaluation.^6^

The video library shows a safe way of providing care and explicitly discusses some differences in practice across the country, and child-specific differences that parents may encounter. The videos have been extensively evaluated by our stakeholder group, and in the formal evaluation with 33 healthcare professionals and 43 parents.^6^ Parents had reported their anxieties about differences in practice, which led to the creation of a specific video to address differences in practice. The videos repeatedly remind families to contact their healthcare professionals if they are unsure about anything raised in the videos, or have further questions.

On our website we have also collated additional resources for families, such as existing videos on the emotional impact of having a child who cannot feed normally, useful Facebook support groups and charities and a short quiz for parents to check their knowledge (https://www.oxstar.ox.ac.uk/more/supporting-parents/introduction). We plan to develop more videos in future to cover topics such as other types of gastrostomy devices and insertion techniques, including the care of gastrojejunal and jejunal tubes.

## Views and responses of parents and healthcare professionals

Here, we summarise feedback received from surveys, interviews and discussions with both parents and clinical staff. We describe our experiences of using the videos in practice. Findings from the formal evaluation of the videos are reported in a separate publication (available open access)^6^: some of the main findings from the evaluation are described briefly below.

Feedback from families indicates that the videos empower families and reduce the fear, especially at the start of their journey. Parents greatly value learning from families in the videos as well as healthcare professionals and seeing real families caring for their children at home. Parents appreciated the normalising effect of seeing care demonstrated in the home environment and the clear step-by-step explanations and demonstrations. A key advantage of videos over verbal information from clinicians is that videos can be re-watched as a refresher as needed: it can be difficult to retain information given in face-to-face training, especially when families are anxious or stressed, such as when their child is having surgery. Families tell us videos are particularly valuable when access to information from healthcare professionals can be difficult, such as evenings, night time or weekends.

Parents and clinicians thought that families who watched the videos before their appointment or face-to-face training would be able to ask better questions, since they have already developed a baseline knowledge. Clinical staff suggested that the videos would potentially reduce demand on community and hospital teams, when families are empowered to manage minor problems themselves. Clinicians can send specific videos to families, which may avoid the need for some home visits for common problems and concerns, or can simply provide families with the reassurance they need. Box 1 provides some quotes from families and healthcare professionals who have used the videos. These quotes are illustrative—a full thematic analysis on the qualitative data is available in the formal evaluation.^6^

Box 1 **Quotes from evaluation of videos**

**Quotes from families**

- "It felt like we were having a consultation/conversation in person. Having videos was dramatically better than having only a printed leaflet."
- "We now feel like we understand the subject and are in a much better position to ask relevant questions in clinic."
- "I think the balance between parents/carers having first-hand experience and also clinicians is really important and done very well."
- "This was so normalising and reassuring. It helped us to imagine our lives when our daughter has her [gastrostomy] button in place. They were incredibly empowering."
- "I like that parents were used in this who have experience—it’s reassuring for new parents to see how normal this new normal is."

**Quotes from healthcare professionals**

- "Parents and carers like to be able to access information in a way that they won’t feel judged and that they can re-watch multiple times. Having videos means that they will not have to contact the community teams/hospital teams for simple questions and this will undoubtedly help to empower them."
- "A really great idea that will be extremely useful for parents to understand what a button is and how to care for it. Parents that might be worried about having one fitted, hopefully these videos will help put the parents’ minds at rest."
- "The information is well balanced, and it acknowledges risks and challenges while still being reassuring. I like that it doesn’t offer false reassurance."
- "The leaflets we have currently are pants, and this will be much better. We have also had difficulties due to recommissioning of CCNs [Community Children’s Nurses] in getting them to see and show information to the families in advance of the first appointment, which historically has been really helpful. I suspect this will be an excellent resource."
- "The videos would be helpful for parents to get an in-depth understanding of the operation and will really help with gaining informed consent."

## Integration of the video library into routine care

The video library is not intended as a substitute for face-to-face training but as an additional resource to support families emotionally and practically. Parents still need initial face-to-face training and clinicians to contact in the event of difficulties. The videos are there to serve as a source of information and reassurance for families, available 24 hours a day, 7 days a week (unlike specialist clinical teams).

**Figure 3:**
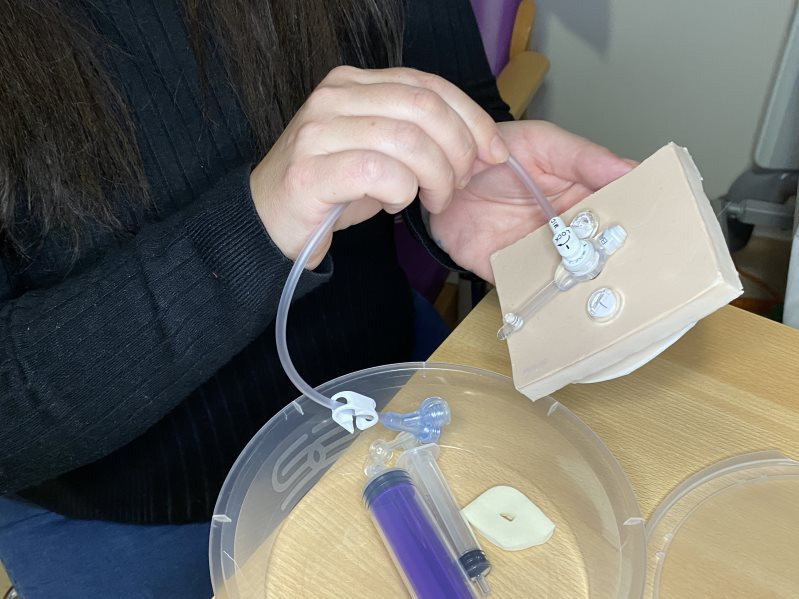
Photo of a parent practising with the box of equipment while in hospital for their child's gastrostomy surgery.

When implementing the videos into routine practice, it is important to view them as one part of a wider package of training and support for families. In addition to the videos, we are also using three-dimensional(3D)-printed models with parents during the hospital admission for surgery. The models are designed to familiarise parents with what the equipment looks and feels like, and enable families to get hands-on practice before performing procedures on their child and build their confidence. Simulation practice is advocated internationally for training families.^7^ The models complement the videos and the existing face-to-face teaching families receive (see figure 3).

In order to gain the full benefits of the resources, it is vital that all organisations and professionals involved in training and supporting families with gastrostomies are aware of the videos and can signpost families to them. For example, paediatricians or dieticians referring a child for a gastrostomy can direct families to the introductory videos, which can help inform and reassure them before they attend their preoperative appointment. Figure 2 illustrates how different topics fit within the patient pathway. Box 2 gives further advice on how to implement the videos in practice.

Box 2 **Advice for implementing the resources into routine practice across a region**

- We are currently integrating the video library into routine clinical practice in our region. If you are considering this, we recommend that you first explore the video library with some families you support as well as with clinical staff from the community and hospital.
- These videos should never be used as a replacement for face-to-face training but as an additional source of support.
- The videos show a safe way of doing things and have been rigorously evaluated by clinicians and families from across the UK. However, there are small differences in practice between what is advised between different regions and organisations, and some child-specific differences. However, the underlying principles of gastrostomy care are universal and the videos will serve as a useful baseline knowledge for families and help support them emotionally.
- You may want to consider sending families some additional written information to accompany the videos with guidance specific to their child. More information about differences in practice are discussed in a short video in the series: https://www.oxstar.ox.ac.uk/more/supporting-parents/differences-in-practice-and-evaluation-of-videos.
- Make all relevant organisations in your region aware of the videos and resources, for example, the surgical team at hospital, community children’s nursing (CCN) teams, dieticians, paediatricians, respite services, special schools, school nurses, etc.
- Share the videos among key networks (eg, clinical networks, any local support groups or Facebook groups for children with specialist needs in your area) and identify key stakeholders who can help disseminate the resources (this may include some parent representatives).
- Ensure each team is aware of which videos will be most important to signpost families to at which time point (see figure 2). CCN teams, for example, should be familiar with the troubleshooting videos and videos on changing a gastrostomy button. Our website also links to some pre-existing troubleshooting videos produced by others.
- Assess whether your region needs further resources to support hands-on practice. For example, in our region, we have purchased three-dimensional(3D)-printed models for helping families get more hands-on practice before performing procedures on their child, and books to explain what a gastrostomy is to children and siblings. For more information, see: https://www.oxstar.ox.ac.uk/more/supporting-parents/your-childs-gastrostomy.
- Consider how the videos might support staff. In our experience, the videos can be informative for students or clinicians new to gastrostomy care, however they are not designed to provide comprehensive training to staff. Specific videos, such as the video on managing over-granulation by the specialist nurse, can be valuable education for non-specialists.

## Conclusions

If families are not given adequate training and support, families feel anxious and isolated, children can come to harm, and there are more callouts to community teams and visits to Accident & Emergency (A&E), which causes more strain on the healthcare system. Our resources empower families and can help to reduce demand on healthcare services and improve outcomes for children, through better, safer care at home. The resources are unique in that they have been coproduced with families, and are designed to support families at different time points across their journey, not just the immediate postoperative period. The videos are designed by (and feature) a multidisciplinary group of hospital and community-based clinicians to provide an integrated approach to training and support. There are many benefits of online resources, especially in light of ongoing restrictions on face-to-face care as a result of COVID-19 and growing pressures on health services. Providing more information to families before clinics/procedures helps families to get more out of the face-to-face time with surgeons and specialist nurses.

Similar support packages coproduced with families and a multidisciplinary group of healthcare professionals are needed for other medical procedures that families perform at home. The videos and resources are freely available to any parent or clinical team. The resources are available here (and are searchable on YouTube): https://www.oxstar.ox.ac.uk/more/supporting-parents/watch-the-videos. QR codes linking to the resources are available in online supplemental file 1.

10.1136/flgastro-2022-102181.supp1 Supplementary data

## Data Availability

Data sharing not applicable as no datasets generated and/or analysed for this study.
