# Supplementary material for: Coproduced resources to support parents caring for children with gastrostomies
Source: Frontline Gastroenterol. 2022 Aug 25;14(2):144–8. doi: 10.1136/flgastro-2022-102181 (PMC9933587; doi:10.1136/flgastro-2022-102181)
Supplement: Supplementary data [file flgastro-2022-102181supp001.pdf]

BMJ Publishing Group Limited (BMJ) disclaims all liability and responsibility arising from any reliance placed on this supplemental material which has been supplied by the author(s)

Supplementary File 1: QR Codes

The use of QR codes is an easy way to quickly access online materials. QR codes can be put on posters, e.g. put on the walls in hospitals/clinics, emailed to families or included on patient information booklets or leaflets. Here are some QR codes for linking to our gastrostomy resources to support families.

QR code for website with resources and videos for families on gastrostomy care

QR code for YouTube playlist for gastrostomy videos

*QR codes can be easily generated online for free to direct people to specific videos as needed.
